# CpG islands or CpG clusters: how to identify functional GC-rich regions in a genome?

**DOI:** 10.1186/1471-2105-10-65

**Published:** 2009-02-20

**Authors:** Leng Han, Zhongming Zhao

**Affiliations:** 1 State Key Laboratory of Genetic Resources and Evolution, Kunming Institute of Zoology, Chinese Academy of Sciences, Kunming, Yunnan 650223, PR China; 2 Virginia Institute for Psychiatric and Behavioral Genetics, Virginia Commonwealth University, Richmond, VA 23298, USA; 3 Graduate School, Chinese Academy of Sciences, Beijing 100039, PR China; 4 Center for the Study of Biological Complexity, Virginia Commonwealth University, Richmond, VA 23284, USA

## Abstract

**Background:**

CpG islands (CGIs), clusters of CpG dinucleotides in GC-rich regions, are often located in the 5' end of genes and considered gene markers. Hackenberg *et al*. (2006) recently developed a new algorithm, CpGcluster, which uses a completely different mathematical approach from previous traditional algorithms. Their evaluation suggests that CpGcluster provides a much more efficient approach to detecting functional clusters or islands of CpGs.

**Results:**

We systematically compared CpGcluster with the traditional algorithm by Takai and Jones (2002). Our comparisons of (1) the number of islands versus the number of genes in a genome, (2) the distribution of islands in different genomic regions, (3) island length, (4) the distance between two neighboring islands, and (5) methylation status suggest that Takai and Jones' algorithm is overall more appropriate for identifying promoter-associated islands of CpGs in vertebrate genomes.

**Conclusion:**

The generation of genome sequence and DNA methylation data is expected to accelerate greatly. The information in this study is important for its extensive utility in gene feature analysis and epigenomics including gene prediction and methylation chip design in different genomes.

## Background

CpG islands (CGIs) are clusters of CpG dinucleotides in GC-rich regions. They are often associated with the 5' end of genes and considered gene markers [1]. Methylation of promoter-associated CGIs plays an important role in gene regulation and carcinogenesis. Because of the functional importance, multiple algorithms have been available for identifying CGIs in a sequence. Traditional algorithms are based on three sequence parameters (length, GC content, and ratio of the observed over the expected CpGs (Obs_CpG_/Exp_CpG_)) that were originally proposed by Gardiner-Garden and Frommer in 1987 [2] and later revised by Takai and Jones [3] and others. These algorithms have been widely used in the identification of CGIs in numerous studies. Among these algorithms, Takai and Jones' [3] stringent algorithm seems to outperform the others because it can effectively exclude short interspersed elements such as *Alu *and it can identify CGIs that are more likely associated with the 5' regions of genes [3].

Recently, Hackenberg *et al*. [4] developed a new algorithm, namely CpGcluster. CpGcluster does not employ the three parameters typically used in traditional algorithms, rather, it detects clusters of CpGs (i.e., CpG clusters) by statistical significance based on the physical distance between neighboring CpGs on a chromosome [4]. To save the space and to compare the CGIs by Takai and Jones' algorithm, we abbreviate CpG clusters as "CGCs" hereafter. Both CGIs and CGCs represent the islands of CpGs in a genome. Their evaluation claimed a better performance of CpGcluster due to its better benchmark, minimal overlap with *Alu*, and higher degree of overlap with promoter and phylogenetic conserved elements. Here we performed an extensive evaluation of the two algorithms (Takai and Jones' algorithm and CpGcluster) in two model organisms (human and mouse) and demonstrated that Takai and Jones' algorithm has an overall better performance in the identification of CGIs in vertebrate genomes.

## Results and discussion

### CGIs versus CGCs: statistics in the human and mouse genomes

Table 1 shows the statistics of CGIs by Takai and Jones' algorithm and CGCs by CpGcluster in the human and mouse genomes. The number of CGCs is remarkably larger than that of CGIs. In the human genome, there are 198,702 CGCs, 5.3 times the number of CGIs (37,729). Similarly, in the mouse genome, the number of CGCs (121,885) is 5.7 times that of CGIs (21,326). The characteristics of CGIs and CGCs are different too. On average, CGIs are much longer than CGCs. In the human genome, CGIs have an average length of 1,090 bp, while CGCs have an average length of only 273 bp. A similar large difference was observed in the mouse genome (1,045 versus 318 bp). This large difference is mainly due to CpGcluster's independence on the minimum length of a cluster. Contrast to the length, CGCs have on average a higher GC content and Obs_CpG_/Exp_CpG _ratio than CGIs (Table 1), reflecting that CpGcluster tends to select shorter but CpG-richer regions.

**Table 1:** Statistics and distribution of CGIs and CGCs in the human (NCBI build 36) and mouse genomes (NCBI build 37)

	Human		Mouse	
	
	CGIs (%^a^)	CGCs (%^a^)	CGIs (%^a^)	CGCs (%^a^)
Genome length (bp)	2.86 × 10^9^	2.86 × 10^9^	2.61 × 10^9^	2.61 × 10^9^
# CGIs/CGCs	37,729	198,702	21,326	121,885
Coverage (%)^b^	1.44	1.90	0.85	1.48
Length (bp)	1,090 ± 717	273 ± 246	1,045 ± 519	318 ± 297
GC content (%)	60.61 ± 5.06	63.78 ± 7.50	60.0 ± 4.0	61.4 ± 10.0
Obs_CpG_/Exp_CpG_	0.717 ± 0.082	0.855 ± 0.265	0.730 ± 0.093	0.949 ± 0.426
Promoter regions	13,196 (35.0)	29,156 (14.7)	10,942 (51.3)	19,791 (16.2)
TSSs	15,106 (40.0)	21,741 (10.9)	12,175 (57.1)	16,675 (13.7)
Genic regions	24,841 (65.8)	104,924 (52.8)	15,541 (72.9)	63,555 (52.1)
Intergenic regions	12,888 (34.2)	93,778 (47.2)	5,785 (27.1)	58,330 (47.9)
				
8-bp CGCs				
Sub-total		232		775
Promoter regions		12 (5.2^c^)		13 (1.7^c^)
Intergenic regions		144 (62.1^c^)		439 (56.6^c^)

^a^Proportion (%) of CGIs or CGCs in the genomic region over the total number of CGIs or CGCs in the genome.

^b^Proportion (%) of the genome sequence covered by CGIs or CGCs.

^c^Proportion (%) of the CGCs in the promoter regions or intergenic regions among the total 8-bp CGCs.

### CGIs versus CGCs: evaluation on gene markers

The main interest in finding islands of CpGs is that they may serve as gene markers [1]. Mammalian genomes have similar sizes (2.0 – 3.0 Gb) and similar numbers of annotated genes (20,000 – 30,000) [5]. Surprisingly, the number of CGCs in the human genome is ~8 times that of human genes and the number of mouse CGCs is ~4 times that of mouse genes. In contrast to the CGCs, the total number of human CGIs (37,729) is moderately larger than the number of human genes and the total number of mouse CGIs (21,326) is even in the low range of the estimated number of mouse genes. It is worth noting that the mouse genome has undergone faster CGI loss than the human during genome evolution [6], thus, we observed a smaller number in the mouse genome by both algorithms.

We then examined the distribution of CGIs or CGCs in different genomic regions including promoter, genic and intergenic regions (see Methods). Among the 37,729 human CGIs, 35.0% were mapped to the promoter regions. However, only 14.7% of the 198,702 human CGCs were mapped to the promoter regions (Table 1). Similarly, we found 51.3% of mouse CGIs but only 16.2% of mouse CGCs mapped to the promoter regions. We had a similar observation when we examined the coverage of islands of CpGs with the transcriptional start sites (TSSs). For example, we observed 40.0% of the human CGIs but only 10.9% of human CGCs covering TSSs (Table 1). Most human CGCs (85.3%) are located outside of the promoter regions and about half are in the intergenic regions.

It is interesting to examine the short CGCs identified by CpGcluster. The shortest CGCs were found to be 8 bp in both the human and mouse genomes. For the 8-bp CGCs, we counted a total of 232 times in humans and 775 times in mice. Hackenberg *et al*. suggested that the very short islands might be functional because they likely overlap with the promoter regions. However, even this is true, we found only few short CGCs (12 counts of human 8-bp CGCs, 5.2%; 13 counts of mouse 8-bp CGCs, 1.7%) are located in the promoter regions. In fact, the majority of them are in the intergenic regions (Table 1). Furthermore, we found all these 8-bp CGCs are octamer CGCGCGCG. Our preliminary search in the TRANSFAC and JASPAR databases indicates that this octamer is rarely part of the regulatory motifs. Similar distribution was observed for CGCs whose length is ≤ 50 bp (data not shown). These results indicate that short CGCs may not serve as markers for genes.

### CGIs versus CGCs: length distribution

The main difference between the two algorithms is that Takai and Jones' algorithm requires a minimal length but CpGcluster does not. The shortest CGCs have only 8 bp compared to the minimum length of 500 bp by Takai and Jones' algorithm. Here we compared the length distribution of CGCs and CGIs in the human and mouse genomes. As expected, the length distribution of the two algorithms is remarkably different. In humans, most CGCs are in a range of 50 – 400 bp long (83.6%), while only 9.8% of CGCs are longer than 500 bp. For those long CGCs (≥ 500 bp), their number distribution is not strongly different from that of CGIs (Additional file 1).

**Figure 1:**
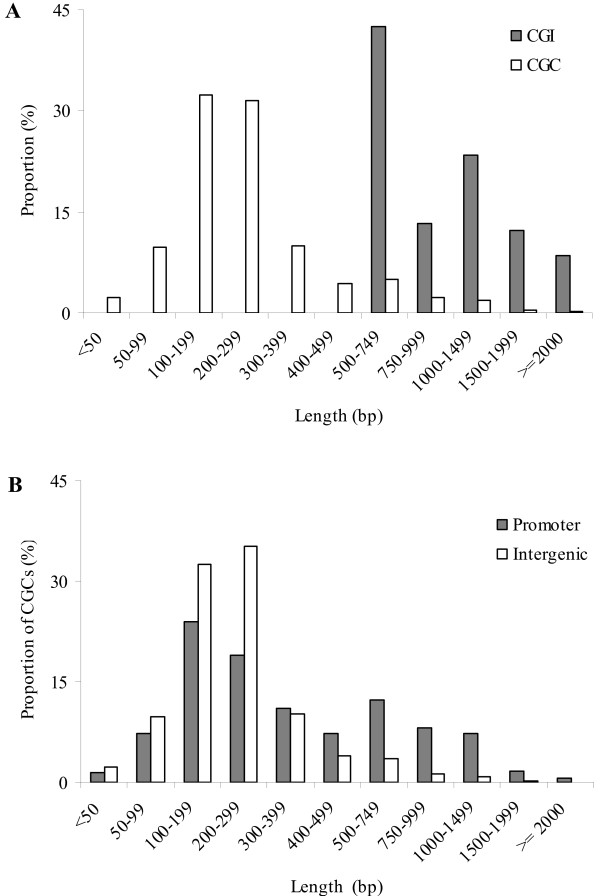
**Length distribution of CGIs or CGCs in the human genome**. (**A**) CGIs versus CGCs. (**B**) For CGCs, promoter regions versus intergenic regions.

Ioshikhes and Zhang [7] found that the CGIs overlapping with TSS are much longer than those outside the gene environment. A similar pattern was observed for CGCs [4]. Here we examined and compared the length distribution for promoter-associated CGCs and intergenic CGCs. When CGCs are shorter than 300 bp in the human genome and 400 bp in the mouse genome, the proportion of promoter-associated CGCs is smaller than that of intergenic CGCs, indicating that those short CGCs are more likely in the intergenic regions rather than in the promoter regions (Figure 1B, Additional file 1). Conversely, when the length is > 500 bp, the proportion of promoter-associated CGCs is noticeably larger than that of intergenic CGCs in both the genomes. The result indicates that the length is an important factor in identifying islands of CpGs that are associated with the promoter regions and, thus, effectively reducing the false positives.

### Multiple CGCs or CGIs in a promoter region

Because the number of CGCs is remarkably larger than that of genes in both the human and mouse genomes, it is necessary to examine whether multiple CGCs or CGIs are located in a genic or promoter region. Among the 24,810 human genes extracted from the NCBI Refseq database, we found that 9,387 (37.8%) have more than 1 CGC but only 781 (3.2%) have more than 1 CGI. This strong difference was similarly found in the mouse genome (17.4% versus 1.4%).

**Figure 2:**
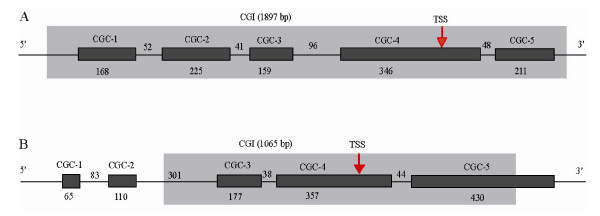
**Multiple short CGCs embedded in one CGI in the promoter region**. Dark box: CGCs identified by CpGcluster. Grey box: CGI identified by Takai and Jones' algorithm. The length of each CGC is labeled below the dark box and the distance between two neighboring CGCs is above the line. The transcription start site (TSS) is marked by an arrow. (**A**) *CAP1*. (**B**) *ADAM33*.

Our further examination revealed that many promoter-associated CGCs are short and "clustered" with short gaps at a locus. In some extreme cases, we found 5–10 CGCs in a promoter region. Figure 2 shows two examples: *CAP1 *and *ADAM33*. *CAP1*, a 32-kb long gene, has five CGCs that lie within its promoter region, but has only one CGI (1,897 bp) identified by Takai and Jones' algorithm. These five CGCs are embedded in that CGI, separated by 52, 41, 96 and 48 bp, respectively (Figure 2A). For *ADAM33*, a 14-kb long gene, we identified five CGCs that lie within or overlap with its promoter region where only one CGI (1,065 bp) was identified (Figure 2B). Three of these CGCs (CGC3, CGC4 and CGC5) are nearly embedded in the CGI. The other two CGCs (CGC1: 65 bp and CGC2: 110 bp) locate in the upstream of the CGI. Interestingly, both CGC1 and CGC2 are overall within an *Alu *element (*AluSc*), which supports that Takai and Jones' algorithm could effectively exclude the repeats. This reflects that CpGcluster tends to identify small parts of CpG islands and, therefore, results in a substantially inflated number of short CGCs.

**Figure 3:**
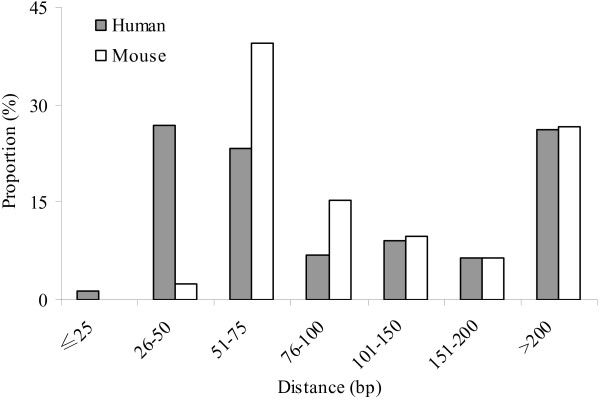
**Distribution of distance between two neighbouring CGCs in the promoter region of a gene**.

We next examined the distance (gap length) between two neighboring CGCs in the promoter regions. Among promoter-associated CGCs, 58.3% of them have distance <100 bp in the human genome; this proportion is 57.2% in the mouse (Figure 3). Moreover, 26.1% of human CGCs and 26.7% of mouse CGCs are separated more than 200 bp. Of note, Takai and Jones' algorithm requires CGIs to be separated at least 100 bp, otherwise they would be merged [3]. When the distance is >100 bp, the proportions are similar in these two genomes (Figure 3). Furthermore, we found similar distribution of distance between two neighboring CGCs in the promoter regions of human-mouse homologous genes (Additional file 2). This again suggests that the length is an important factor.

### CGIs versus CGCs: evaluation on methylation status in the promoter regions

Functional GC-rich regions are often unmethylated, which is an important feature in the regulation of gene expression. Ideally, a computationally identified CGI or CGC is associated with a promoter region that is unmethylated or hypomethylated. To evaluate the performance of these two algorithms on predicting islands of CpGs that are unmethylated or hypomethylated, we obtained 7,684 genes with methylation status in their promoter regions from Weber *et al*. [8]. We calculated sensitivity, specificity, accuracy and correlation coefficient (see Methods). Although CpGcluster has a slightly better sensitivity (CGCs: 0.96; CGIs: 0.93) and accuracy (CGCs: 0.91; CGIs: 0.90), its specificity (0.42) is noticeably lower than that (0.62) of Takai and Jones' algorithm. When all factors combined, we found a larger correlation coefficient by Takai and Jones' algorithm (0.48) than CpGcluster (0.40).

## Conclusion

In this study, we systematically compared two representative algorithms: Takai and Jones' algorithm and the distance-based algorithm aiming for identifying functional CpG clusters. Our comparison of the number of islands versus the number of genes in a genome, the distribution of islands in different genomic regions, the length distribution, the distance between two neighboring islands in the promoter regions, and methylation status suggests that Takai and Jones' algorithm is overall more appropriate for identifying promoter-associated islands of CpGs. Although CpGcluster can uniquely identify some short CpG clusters that are functional, its high false positive rate strongly limits its utility in genome-wide or chromosome-wide searching promoter-associated CpG clusters in vertebrate genomes. Since CpG islands represent an important gene feature and are expected to be used extensively in gene prediction, gene feature analysis, and epigenetic and epigenomic projects, the information in our study is fundamental.

## Methods

### Identification of CGIs and CGCs

We used the stringent criteria in Takai and Jones [3] to search CGIs: length ≥ 500 bp, GC content ≥ 55% and Obs_CpG_/Exp_CpG _≥ 0.65. We used default cutoffs in CpGcluster in Hackenberg *et al*. [4] to search CGCs: the median distance and *P*-value = 10^-5^.

### Genome sequences and gene annotations

We downloaded the assembled human and mouse genome sequences and their gene annotation files from the NCBI database (human build 36, mouse build 37) [9]. A gene may have more than one transcript. We determined the location of gene's TSS based on the transcript that extends towards 5' most to avoid the influence of other genic environment. Hackenberg *et al*. examined the CGIs or CGCs that overlapped with the TSS. However, mammalian genes tend to initiate transcription at multiple alternative start sites across a region, rather than a single well-defined site [10]. Thus, in this study we examined the CGIs or CGCs that lie within or overlap with the promoter regions. A promoter region was defined -1,500 to + 500 bp around the TSS. Separately, we obtained 563,593 and 213,920 distinct TSSs in the human and mouse genomes from the DBTSS database [11] as a gene may have multiple distantly-dispersed TSSs [12]. We used a pipeline developed by Jiang and Zhao [13] to identify genic and intergenic regions in the human and mouse genomes. We retrieved 16,587 human-mouse homologous genes from the HomoloGene database (build 61) [14].

### Methylation status of CGIs/CGCs in the promoter regions

Weber *et al*. [8] recently examined and measured methylation status of ~16,000 promoters in human WI38 primary lung fibroblast. They defined a promoter being hypermethylated when its 5 mC log2 ratio was >0.4, otherwise hypomethylated. We used this dataset to evaluate methylation status of CGIs/CGCs in the promoter regions. We identified 7,684 genes with methylation status in their promoter regions including 697 hypermethylated and 6,987 hypomethylated promoters.

To evaluate the performances of two algorithms on predicting functional islands or clusters, we examined the methylation status in promoter-associated CGIs or CGCs. We calculated the sensitivity (Sn), specificity (Sp), accuracy (Ac) and correlation coefficient (Cc). The equations are:

(1) *Sn *= *TP*/(*TP *+ *FN*)

(2) *Sp *= *TN*/(*TN *+ *FP*)

(3) *Ac *= (*TP *+ *TN*)/(*TP *+ *TN *+ *FP *+ *FN*)

(4) $$Cc=\left(\left(TP\times TN\right)-\left(FN\times FP\right)\right)/\sqrt{\left(TP+FN\right)\times \left(TN+FP\right)\times \left(TP+FP\right)\times \left(TN+FN\right)}$$

where TP are true positives (hypomethylated promoters containing CGIs or CGCs); TN are true negatives (hypermethylated promoters without detecting CGIs or CGCs); FP are false positives (hypermethylated promoters containing CGIs or CGCs); and FN are false negatives (hypomethylated promoters without detecting CGIs or CGCs).

## Authors' contributions

LH and ZZ prepared the data, carried out the analysis and wrote the manuscript.

## Supplementary Material

Additional file 1 **Comparison of the length distribution of CGIs and CGCs.** This file includes the comparison of the length distribution of CGIs and CGCs in the human and mouse genomes. Click here for file

Additional file 2 **Figure S2.** Figure S2 displays the distribution of distance between two neighboring CGCs in the promoter region of human-mouse homologous genes. Click here for file
