# Supplementary material for: CpG islands or CpG clusters: how to identify functional GC-rich regions in a genome?
Source: BMC Bioinformatics. 2009 Feb 20;10:65. doi: 10.1186/1471-2105-10-65 (PMC2652441; doi:10.1186/1471-2105-10-65)
Supplement: Additional file 1 — Comparison of the length distribution of CGIs and CGCs. This file includes the comparison of the length distribution of CGIs and CGCs in the human and mouse genomes. [file 1471-2105-10-65-S1.pdf]

Additional file 1 Comparison of the length distribution of CGIs and CGCs

Figure S1. Length distribution of CGIs and CGCs. (**A**) Number of CGIs versus CGCs in the human genome. (**B**) Number of CGCs in the promoter regions versus intergenic regions in the human genome. (**C**) Proportion of CGIs versus CGCs in the mouse genome. (**D**) Number of CGIs versus CGCs in the mouse genome. (**E**) Proportion of CGCs in the promoter regions versus intergenic regions in the mouse genome. (**F**) Number of CGCs in the promoter regions versus intergenic regions in the mouse genome.

A

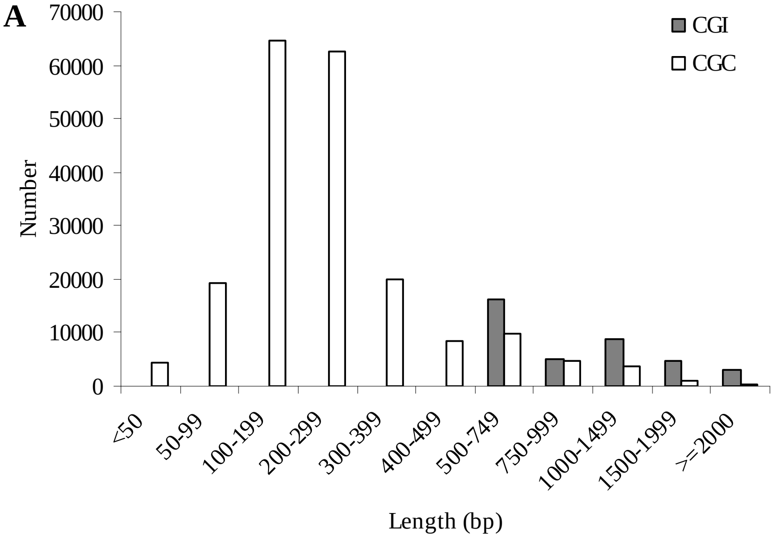

B

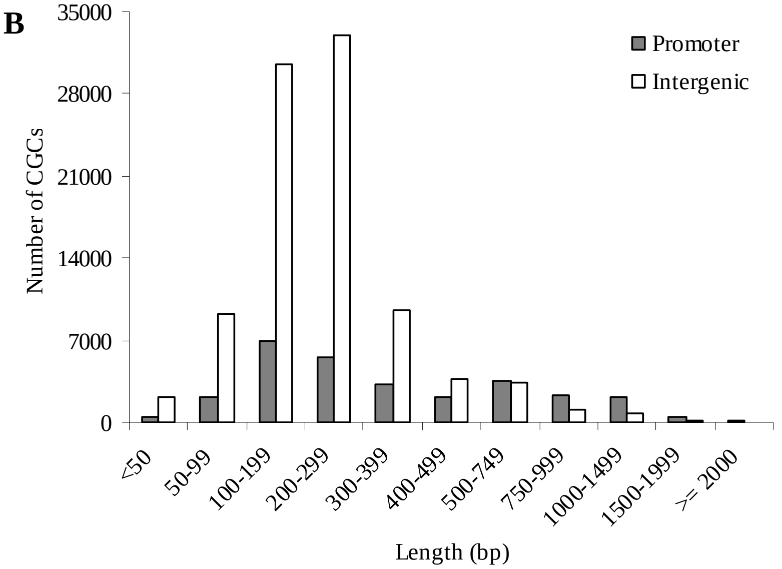

C

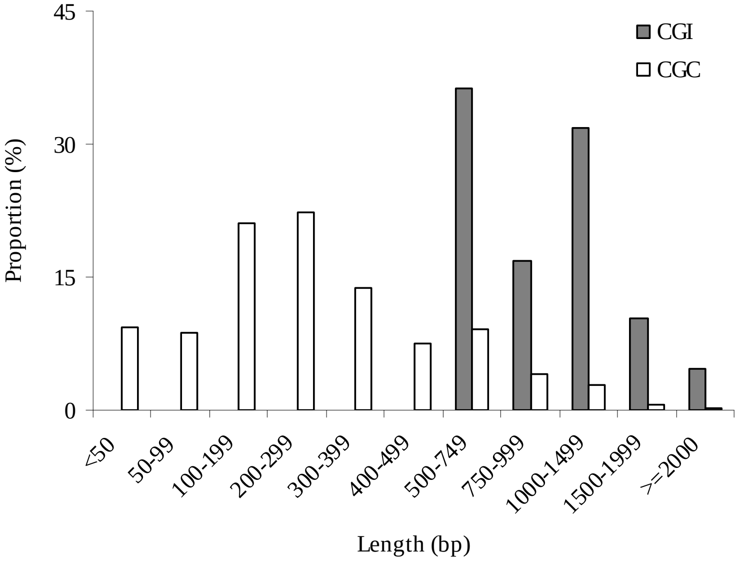

45
30
15
0
Proportion (%)
CGI
CGC
<50
50-99
100-199
200-299
300-399
400-499
500-749
750-999
1000-1499
1500-1999
>=2000
Length (bp)

D

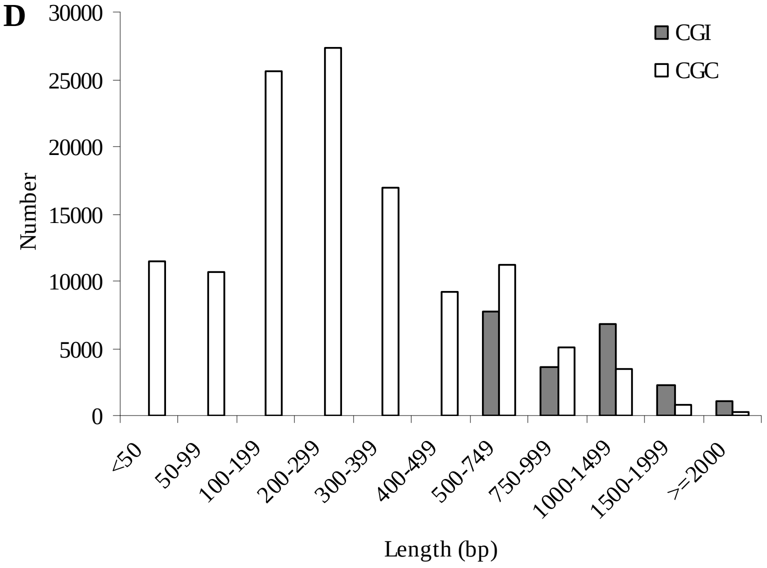

30000
25000
20000
15000
10000
5000
0
Number
CGI
CGC
<50
50-99
100-199
200-299
300-399
400-499
500-749
750-999
1000-1499
1500-1999
>=2000
Length (bp)

E

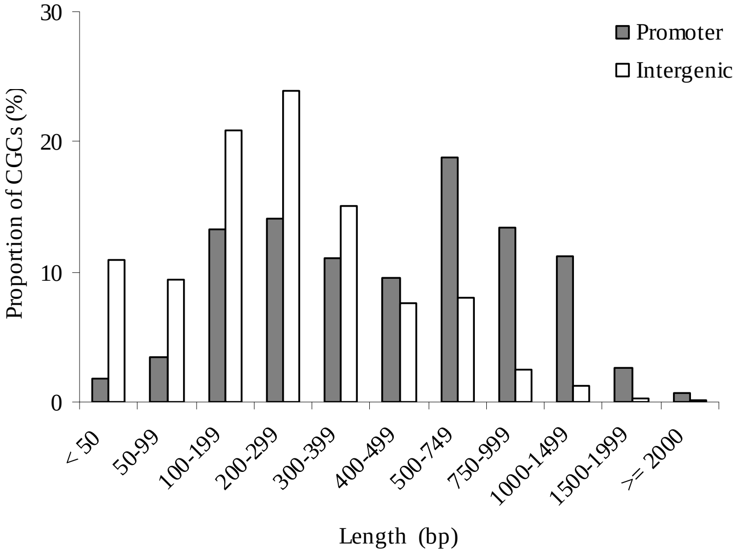
Promoter
Intergenic
Proportion of CGCs (%)
30
20
10
0
< 50
50-99
100-199
200-299
300-399
400-499
500-749
750-999
1000-1499
1500-1999
>= 2000
Length (bp)

F

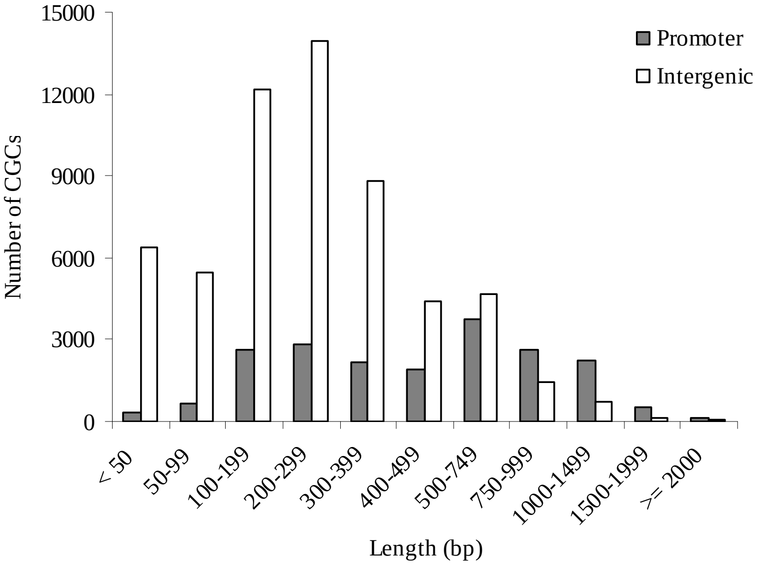
Promoter
Intergenic
Number of CGCs
15000
12000
9000
6000
3000
0
< 50
50-99
100-199
200-299
300-399
400-499
500-749
750-999
1000-1499
1500-1999
>= 2000
Length (bp)
