# Supplementary material for: CpG islands or CpG clusters: how to identify functional GC-rich regions in a genome?
Source: BMC Bioinformatics. 2009 Feb 20;10:65. doi: 10.1186/1471-2105-10-65 (PMC2652441; doi:10.1186/1471-2105-10-65)

Additional file 2

Figure S2. Distribution of distance between two neighboring CGCs in the promoter region of human-mouse homologous genes.

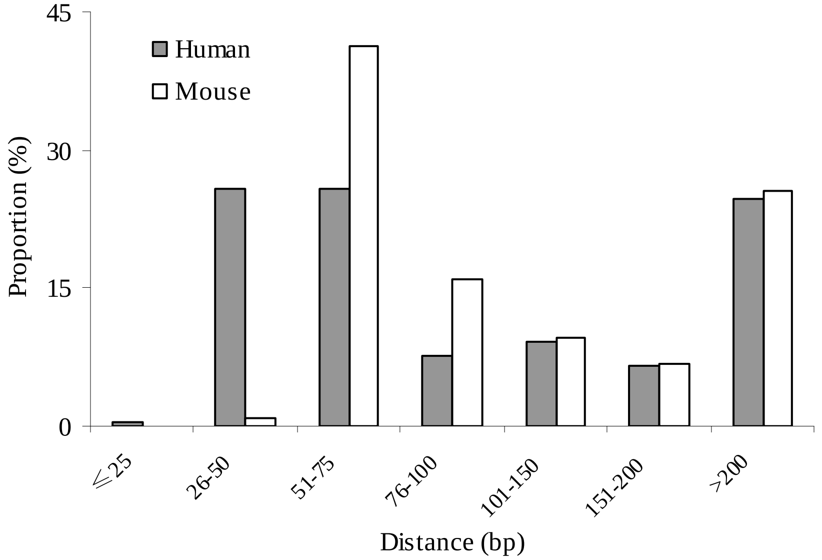

Supplement: Additional file 2 — Figure S2. Figure S2 displays the distribution of distance between two neighboring CGCs in the promoter region of human-mouse homologous genes. [file 1471-2105-10-65-S2.pdf]
